# The Effect of Combining Transcranial Direct Current Stimulation Treatment and an Exercise Program on Fragility in a Population with Multiple Sclerosis: Cross-Over Design Trial

**DOI:** 10.3390/ijerph191912747

**Published:** 2022-10-05

**Authors:** Inés Muñoz-Paredes, Azael J. Herrero, Rocío Llamas-Ramos, Vicente Rodríguez-Pérez, Jesús Seco-Calvo

**Affiliations:** 1 Faculty of Health Sciences, University of Leon, 24071 León, Spain; 2 Department of Health Sciences, European University Miguel de Cervantes, 47012 Valladolid, Spain; 3 Research Center on Physical Disability, ASPAYM Castilla y León, 47008 Valladolid, Spain; 4 Department of Physical Therapy, University of Salamanca, 37007 Salamanca, Spain; 5 Institute of Biomedicine (BIOMED), University of Leon, 24071 León, Spain; 6 Physiology Department, University of the Basque Country, 48940 Leioa, Spain

**Keywords:** depression, functional mobility, balance, physical training

## Abstract

Background: The relationship between fragility and neurological diseases is extensive and affects many patients with multiple sclerosis (MS), whose risk factors are related to fragility. Objective: To study the effects of exercise and transcranial direct current stimulation (tDCS) in MS patients with fatigue from four dimensions: functional mobility, balance, fatigue, and depression. Methods: A total of 12 patients who belonged to two associations of people with physical disabilities participated. Functional mobility, depression, balance, and fatigue were assessed before and after the intervention. Transcranial direct current stimulation and the exercise program were carried out over a 4-week period with a wash-out period of 5 months. Results: After the application of tDCS, we found significant improvements in balance (*p* < 0.05, g = 0.632), depression (*p* < 0.05, g = 0.402), functional mobility (*p* < 0.05, g = 0.297), and fatigue (*p* < 0.05, g = 0.526). After the exercise program, significant improvements were shown in balance (*p* < 0.01, g = 0.418), depression (*p* < 0.001, g = 0.540), functional mobility (*p* < 0.01, g = 0.262), and fatigue (*p* < 0.01, g = 0.742). Two-way mixed-measures ANOVA showed that all variables improved in both groups, with significant differences over time but not between groups. Secondary analysis showed significant correlations between balance and functional mobility (r = 0.671, *p* = 0.017), depression and fatigue (r = 0.586, *p* = 0.044) and between intensity of rehabilitation and balance (r = 0.622, *p* = 0.031). CONCLUSION: Participating in an exercise program and receiving tDCS separately improved the variables of depression, balance, mobility, and fatigue.

## 1. Introduction

Fragility is a multidimensional condition of increased vulnerability to stress and/or reduced reserves, which generates a risk of adverse health in those who are affected by it [1,2]. The fragility cycle, described by Fried [3], refers to a negative cycle of malnutrition, decreased strength and exercise tolerance, and decreased total energy expenditure, which can be activated or enhanced by any adverse event.

The relationship between fragility and neurological diseases has been the subject of numerous studies in the field of neuroscience. It has been determined that each neurodegenerative disease appears to primarily affect certain subsets and neuronal populations [1]. One of them is multiple sclerosis (MS), the main cause of neurological disability in young adults in the Western world. Although the cause of this disease is unknown, some risk factors are known and are related to fragility, such as weight alteration and a low level of physical activity [1,4].

Physical inactivity levels in the MS population are different from those in the normal population [5]. One of the most prevalent symptoms in these patients is mobility alterations, present in 80% of MS cases. In some cases, these disturbances are intensified by alterations in balance, which, in turn, are related to an increased risk of falls. Depression, with a prevalence of 24% in this population, is associated with the state of fragility. In fact, if this cognitive impairment is recognized too late, the problem of reversing the fragility becomes much more complicated [6].

Exercise programs have been recommended in recent years due to their potential to reduce the degenerative process by modifying the anti-inflammatory effect of the disease [7]. Thus, concurrent training is one of the most effective methods for reversing these alterations. The combination of aerobic and strength training produces improvements in mobility, balance, and fatigue and is better tolerated than other types of training [8]. In this sense, the practicing aerobic exercise produces improvements in various cognitive conditions, including depression [9].

On the other hand, a new approach for the management of this type of symptomatology in MS is non-invasive brain stimulation (NIBS), with techniques including transcranial direct current stimulation (tDCS). The effect of this current is focused on modulating brain activity to produce changes in cortical excitability and to generate beneficial effects on fatigue and depression. Some authors, such as Chalah [10], have applied anodal-type tDCS on the left dorsolateral prefrontal cortex (DLPFC), with positive results in fatigue, and on the posterior parietal cortex, improving depression.

Few studies have reported the application of this type of current on motor skills, and none have been reported on DLPFC. Messen et al. [11] applied it on the primary motor area in a single session with an intensity of 1 mA, with no beneficial effects.

Current evidence shows that physical exercise is necessary for maintaining physical and cognitive function in MS patients [7]. However, few studies have assessed physical and cognitive variables in combined training for MS patients with fatigue [11,12,13,14]. In addition, there are no studies of this type that include both exercise and tDCS. It is also important to note that in most of the studies in which this type of current was applied to patients with MS, the number of sessions was very small, and one of them assessed these types of variables jointly. For this reason, it is relevant to study the effects of both treatments (exercise and tDCS) in a population of MS patients with fatigue from four dimensions: functional mobility, balance, fatigue, and depression. Our hypothesis is that subjects who both participate in exercise and receive tDCS will show improvements in the variables analysed.

## 2. Materials and Methods

### 2.1. Study Design and Participants

This cross-over design trial was approved by the legal ethical committee of the University of León (ULE-010-2020). The study followed the principles described in the Declaration of Helsinki. All participants gave written informed consent prior to participation. 

This clinical trial is registered in a WHO-approved public trials registry, the Australian New Zealand Clinical Trials Registry (ANZCTR): https://www.anzctr.org.au/, accessed on, registration number: ACTRN12622000264785 (accessed on 1 February 2020).

A total of 15 subjects participated in the study, including 9 males and 6 females; 3 were excluded. Recruitment of the subjects was carried out at the Palencia headquarters of Aspaym Castilla y León (Spain) and the multiple sclerosis association of Palencia, where the interventions were also performed. 

The inclusion criteria were as follows: a diagnosis of MS, a score indicating the presence of fatigue on the Modified Fatigue Impact Scale (MFIS) [15], the ability to walk at least 20 m without resting, age over 18 years, and good written and spoken Spanish comprehension. Patients were excluded if they presented other diseases that could affect muscle function, or presented a cardiovascular risk profile, respiratory disease, severe pulmonary disease, or other diseases that could interfere in the exercise program.

### 2.2. Interventions

Transcranial Direct Current

The HDCstim stimulator (Newronika, Milán, Italy) was used to apply tDCS: #HS0042/01-13; HDcel: #HE0021/02-13. Direct current was distributed over the scalp with 35 cm^2^ sponge electrodes. During the session, the current was ramped up during the first 15 s to a maximum of 2 mA, which was maintained throughout the stimulation session. The administration of current by a specialised physiotherapist took place during 10 sessions of 20 min duration over a period of 4 weeks. 

Current application points were chosen following the 10-20 EEG system, as it has proven to be a good, low-cost method for localising specific cortical areas. The anode was placed in the left DLPFC region (F3 according to the 10-20 EEG system), while the cathode was placed in the right supraorbital cortex. 

Exercise program

The exercise program, applied by a specialised physiotherapist, consisted of concurrent training (Table 1).

The strength training consisted of circuit training, with 6 exercises involving pushing and pulling exercises for the lower and upper limbs, while pelvic girdle and trunk were worked on. Two circuits, A and B, were developed, so that the subjects could choose one according to whether they found any of the exercises difficult to perform due to mobility. In addition, the repetitions to be performed were set, along with the rest time between exercises and circuits. Each subject began week 1 by performing 2 sessions, on alternate days, of 6 exercises with 15 repetitions each and 2 min of rest between exercises, repeated twice. In week 4, they performed 3 sessions, every other day, of 6 exercises with 10 repetitions each and 3 min of rest between exercises, repeated 3 times.

Aerobic training was increased from 1 session of 10 min in week 1 to 2–3 sessions (depending on the subject’s ability) of 30 min with 5 min of rest between sessions. The intensity was moderate, corresponding to a level of 3–5 on the scale of perceived exertion. A static bike or MOTOmed^®^ kinesiotherapy equipment (RECK-Technik GmbH & Co. KG, Betzenweiler, Germany) was used depending on the participant’s preference.

### 2.3. Procedure

First, the study information sheet was handed out and participants signed the informed consent form. Afterwards, each participant was given a registration form with basic information to verify compliance with the inclusion and exclusion criteria of the study, and to complete the sociodemographic questionnaire. Then, all outcome measures were collected.

Subsequently, transcranial direct current stimulation was applied in 10 sessions of 20 min duration, distributed over 4 weeks. All participants initially received stimulation. After the application of the intervention, fatigue, depression, balance, and mobility were measured again with the questionnaires and tests used at the beginning.

As this was a cross-over design, there was a stabilisation period of 5 months to prevent the results obtained after the first intervention from affecting the second intervention. Following this period, fatigue, depression, balance, and mobility data were collected again, and the concurrent training program was applied for a period of 4 weeks. Finally, fatigue, depression, balance, and mobility data were collected.

### 2.4. Outcome Measures

All outcome measures were collected before commencing with the interventions. After the application of tDCS, the outcome measures of balance, depression, mobility, and fatigue were collected again. Similarly, before and after the application of the exercise program, the outcome measures of balance, depression, mobility, and fatigue were reassessed.

The Beck Depression Questionnaire (BDI-II) [16] is the most specific instrument for measuring depression. It is validated in the MS population and has good psychometric properties for evaluating the severity of depressive symptoms. It proposes the following degrees of depression according to the scores obtained: 0–13, minimal depression; 14–19, mild depression; 20–28, moderate depression; and 26–63, severe depression [17].

The functional mobility test (TUG) is validated in the MS population for the assessment of functional mobility. In addition, it can assess gait and correlates with balance and fall prevention in the elderly. For this test, patients start by sitting in a chair without armrests and are asked to stand up, walk 3 metres, and turn a cone, then sit back down in the chair as fast as possible without running. The score is obtained from the average of 3 attempts [18,19].

To assess balance, we used the Tinetti balance test, which has been validated for the MS population (intraclass correlation R > 0.8). This scale correlates with the Timed Up and Go Test (r = −0.55) in terms of the relationship between balance and risk of falls [20,21].

Clinical, anthropometric, and sociodemographic data were collected on an individual basis, for which a questionnaire was developed to collect data on age, educational level, employment status, presence of diseases, type of sclerosis, years of diagnosis, outbreaks in the last year, intensity of the outbreak, medication used for fatigue, medical recommendations to treat fatigue, attendance at rehabilitation, and exercise habits.

Physical activity was assessed at baseline using the Spanish version of the International Physical Activity Questionnaire Short Form (IPAQ-SF). The validity and reliability of this questionnaire has been studied and tested in different contexts and countries, as well as in different types of populations, including in MS. This questionnaire measures the frequency and duration of vigorous and moderate physical activity and walking during a 7-day period. The respective frequencies and durations are initially multiplied, and the resulting volumes are then multiplied by 8 for vigorous activity, 4 for moderate activity, and 3.3 for walking to obtain METs [22,23].

The assessment of neurological involvement and disability was performed in the first data collection using the Kurtzke Disability Scale (EDSS). It is based on neurological examination findings and consists of 20 grades on a scale from 0 (normal examination) to 10 (death due to MS) with 0.5-point intervals. Patients are evaluated on the basis of the neurological examination and clinical history for each functional system, and then an overall score is obtained taking into account the ability to walk [24,25].

Fatigue was assessed before and after each intervention using the Spanish version of the fatigue questionnaire (MFIS), whose validity and reliability has been studied in different countries. This scale uses a multidimensional approach and consists of 21 items distributed in 3 subscales: physical, cognitive, and psychosocial. The patient responds to each item according to the frequency of symptom occurrence during the last week. The final score ranges from 0 to 84, and a score of 38 is established as the cut-off point to define the presence of fatigue or not [15,26].

### 2.5. Statistical Analysis

The Statistical Package for Social Sciences (SPSS 21, SPSS Inc., Chicago, IL USA) was used for statistical analysis. Descriptive statistics was used in the data analysis to show the data for continuous variables, presented as ± standard deviation (SD), and categorical variables, as frequency (percentage). The normality of the variables was evaluated using the Shapiro–Wilk test, and the result indicated that not all variables met normality, so non-parametric tests were used for statistical calculation.

Several study systems were used to evaluate the results of the intervention: Wilcoxon signed-rank test was used to analyse the results obtained after applying tDCS and the exercise program in relation to the variables balance, depression, functional mobility, and fatigue. Spearman’s correlation (r) was used to determine correlations between the variables of disability, fatigue, and physical activity and the rest of the descriptive variables, with r values showing high (±0.80), moderate (±0.50), and weak (±0.20) differences [27]. The outcomes of the indicators fatigue, functional mobility, balance, and depression were analysed by two-way mixed-measures analysis of variance (ANOVA) with a between-individual factor group (tDCS and exercise) and a within-individual factor time (pre-treatment and post-treatment). Greenhouse-Geisser correction was used when necessary to correct for nonsphericity. In addition, effect size among participants was calculated using partial eta-squared (ƞ^2^p), a measure of effect or size association. Since this measure often overestimates effect size, values were interpreted as follows: 0 < 2p < 0.05 indicates no effect; 0.05 < ƞ^2^p < 0.26 indicates a minimal effect; 0.26 < ƞ^2^p < 0.64 indicates a moderate effect; and ƞ^2^p > 0.64 indicates a strong effect.

Finally, the rehabilitation intensity variables were compared with the primary measurement variables and fatigue using the U-Mann Whitney test. The effect size was calculated to express the magnitude of differences between samples, expressed as Hedges’ g (scale: 0–1). The effect sizes were set as small (0.2–0.5), medium (0.5–0.8), and large (>0.8). [28].

The significance level for all tests was set at *p* < 0.05.

## 3. Results

**Figure 1:**
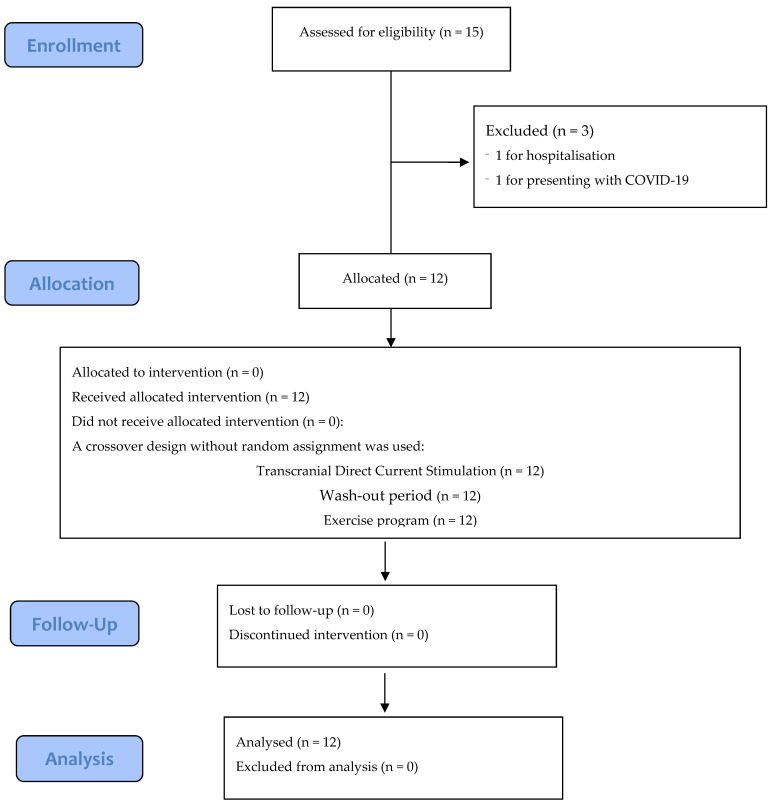
CONSORT flow diagram of this study.

The flow diagram can be seen in Figure 1. Between March 2020 and April 2021, 15 patients participated in the study, and 3 were excluded for the following reasons: one was hospitalised for exacerbation of the disease, one presented with COVID-19, and one underwent a surgical intervention that prevented him from completing the exercise program. The initial characteristics of the participants are shown in Table 2.

We observed that after both tDCS and the exercise program, all subjects had increased percentages in the scales of balance, functional mobility, depression, and fatigue, indicating improvement. The analysis of related samples (Table 3) shows significant improvement in all variables after both treatments. After tDCS, the results were as follows: Tinetti scale: *p* = 0.019, g = 0.632; Beck scale: *p* = 0.013, g = 0.402; and TUG test: *p* = 0.012, g = 0.297. After the exercise program, the results were: Tinetti scale: *p* = 0.004, g = 0.418; Beck scale: *p* = 0.013, g = 0.540; and TUG test: *p* = 0.002, g = 0.262. For fatigue, after tDCS, 50% of subjects had scores below 38 on the MFIS (*p* = 0.028, g = 0.526) and after the exercise program, 58% of subjects had a reduced fatigue score (*p* = 0.003, g = 0.742) (Supplementary Figure S1). It is also interesting to note the change in the type of depression after both interventions, as there was a clear reduction in the intensity of depression, as shown in Supplementary Figure S2.

**Figure 2:**
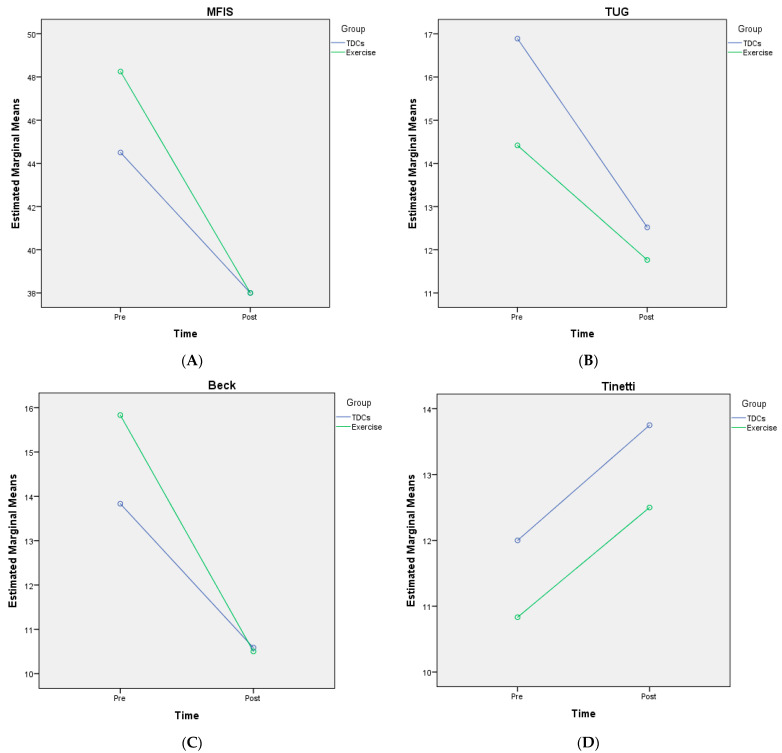
Two-way mixed-measures analysis of variance: (**A**) fatigue questionnaire (MFIS); (**B**) functional mobility test (TUG); (**C**) Beck Depression Questionnaire; (**D**) balance test (Tinetti); mean scores and effectiveness for tDCS and exercise groups before treatment (pre) and after treatment (post).

ANOVA results showed that all variables improved in both groups, with significant differences over time but not between groups (Figure 2). 

MFIS scores were as follows: (mean (SD)): pre-tDCS: 44.5 (9.69); post-tDCS: 38 (13.89); pre-exercise: 48.25 (11.65); post-exercise: 38 (14.85). The analysis of MFIS scores showed that there was nonsphericity and therefore, the Greenhouse–Geisser correction was employed to correct the degree of freedom. The corrected results revealed a significant difference over time (F = 18.01, *p* < 0.0001, η^2^p = 0.45, ε = 0.98), but no interaction between time and group (F = 0.90, *p* = 0.35 η^2^p = 0.39, ε = 0.149). The improvement trend was consistent between the tDCS and exercise groups. There was no significant difference between groups (F = 0.15, *p* = 0.69 η^2^p = 0.07, ε = 0.06) (Figure 2A).

TUG scores were as follows: (mean (SD)): pre-tDCS: 16.88 (17.33); post-tDCS: 12.52 (10.3); pre-exercise: 14.41 (11.14); post-exercise: 11.7 (8.19). The analysis of MFIS scores showed that there was nonsphericity and therefore, the Greenhouse–Geisser correction was employed to correct the degree of freedom. The corrected results revealed a significant difference over time (F = 7.14, *p* = 0.014, η^2^p = 0.25, ε = 0.724), but no interaction between time and group (F = 0.42, *p* = 0.52, η^2^p = 0.19, ε = 0.96). The improvement trend was consistent between the tDCS and exercise groups. There was no significant difference between groups (F = 0.11, *p* = 0.74, η^2^p = 0.05, ε = 0.06) (Figure 2B).

Beck scores were as follows: (mean (SD)): pre-tDCS: 13.83 (8.07); post-tDCS: 10.58 (7.52); pre-exercise: 15.83 (10.47); post-exercise: 10.5 (8.47). The analysis of MFIS scores showed that there was nonsphericity, therefore the Greenhouse–Geisser correction was employed to correct the degree of freedom. The corrected results revealed a significant difference over time (F = 33.89, *p* < 0.0001, η^2^p = 0.60, ε = 1), but no interaction between time and group (F = 1.99, *p* = 0.17, η^2^p = 0.08, ε = 0.27). The improvement trend was consistent between the tDCS and exercise groups. There was no significant difference between groups (F = 0.07, *p* = 0.78, η^2^p = 0.03, ε = 0.05) (Figure 2C).

Tinetti scores were as follows: (mean (SD)): pre-tDCS: 12.83 (2.62); post-tDCS: 13.75 (2.73); pre-exercise: 10.83 (3.97); post-exercise: 12.5 (3.75). The analysis of MFIS scores showed that there was nonsphericity, therefore the Greenhouse–Geisser correction was employed to correct the degree of freedom. The corrected results revealed a significant difference over time (F = 23.02, *p* < 0.0001, η^2^p = 0.51, ε = 0.99), but no interaction between time and group (F = 0.01, *p* = 0.9, η^2^p = 0.01, ε = 0.05). The improvement trend was consistent between the tDCS and exercise group. There was no significant difference between groups (F = 0.85, *p* = 0.36, η^2^p = 0.03, ε = 0.14) (Figure 2D).

Secondary analysis showed significant correlations between balance and functional mobility (r = 0.671, *p* = 0.017), depression and fatigue (r = 0.586, *p* = 0.044), and intensity of rehabilitation and balance (r = 0.622, *p* = 0.031).

## 4. Discussion

To the best of our knowledge, at the time of writing, the present study is the first to address the effect of fragility in MS from the dimensions of functional mobility, balance, fatigue, and depression, comparing the application of tDCS and an exercise program. After the tDCS and exercise program, significant and clinically relevant results were obtained in balance, depression, functional mobility, and fatigue. Moreover, all variables improved in both groups, with significant differences over time. Although the effect size is small for the variables balance and functional mobility after tDCS, and for depression and functional mobility after the exercise program, all correlations except for fatigue after tDCS are strong.

Currently, there are few studies assessing motor performance after tDCS in this population. Some studies have focused on gait and fine motor skills and speed as objectives [11,12,14,29], but none examined functional mobility or balance as we have. Only two studies assessed functional mobility after the application of stimulation [13,30]. Only Pilloni’s study was performed on MS patients, with stimulation of the motor cortex followed by 20 min of aerobic exercise, and significant results were not obtained. Although it has been shown that a single session of anodic tDCS is sufficient in generating improvements at the motor level, it is suggested that the cumulative effect of multiple sessions is necessary to generate the necessary adaptations [11,31].

It is perhaps for this reason that our study obtained significant results for functional mobility and balance, two essential variables related to the risk of falls. It is well known that the predisposition to falls among MS patients is around 56% and that falls cause major limitations in mobility and quality of life. Therefore, an improvement in functional mobility and balance would reduce the incidence of falls and improve the consequent repercussions in the physical, psychological, and social dimensions, as shown by the correlation in our study between the TUG test and the Tinetti scale.

On the other hand, maintaining and increasing muscle power is also important to increase safety during the performance of functional tasks for fragile populations [7,32], so that the implementation of an exercise program such as the one we applied has a significant place in this population. One of the strengths of our study, which was proposed in only a few previous studies, is the implementation of a combined exercise program in a population using scores that determine the presence of fatigue. This type of exercise program was chosen because it has been shown to be effective in the treatment of fatigue, which in turn improves functional mobility, balance, and depression [7]. However, other studies showed no significant improvements in these aspects, which may be due to the varied interventions applied, insufficient intervention periods, or the type of population examined. It is important to highlight that in this aspect, our study complies with the scientific recommendations for designing an exercise program, based on progressive exercise of moderate intensity for MS patients with moderate disability [7,32,33].

Likewise, with regard to the combination of tDCS and physical exercise, recent research supports the potential effects that this combination can have, such as pain regulation in patients with fibromyalgia and improved cognitive abilities in patients with Parkinson’s disease [34,35]. In the MS population, the Pilloni study [13] applied only a single session of tDCS and exercise, thus it does not report significant findings in terms of improved gait and functional mobility. We therefore believe that it would be important in the future to research and study the effects of treatment programs that combine tDCS and physical exercise on MS patients to confirm whether these therapies actually enhance and optimise MS treatment.

Finally, we conclude that our results suggest that the application of an exercise program and of tDCS separately will improve the variables of depression, balance, mobility, and fatigue and that an improvement in fragility can be projected in this group. However, more studies with unified protocols are needed to optimise the results.

## Figures and Tables

**Table 1:** Exercise program schedule.

	Week 1	Week 2	Week 3	Week 4
**Strength**	2 Sessions (A,B)	2 Sessions (A,B)	3 Sessions (A,B, A/B to choose)	3 Sessions (A,B, A/B to choose)
	6 Exercise/circuit	6 Exercise/circuit	6 Exercise/circuit	6 Exercise/circuit
	15 Repetitions (2 min rest)	15 Repetitions (2 min rest)	10 Repetitions (3 min rest)	10 Repetitions (3 min rest)
	2 Circuits (3 min rest)	2 Circuits (3 min rest)	3 Circuits (5 min rest)	3 Circuits (5 min rest)
**Endurance**	1 Session	2 Sessions	2 Sessions	2–3 Sessions
	10 min	15 min	10 + 10 min (5 min of rest	15 + 15 min (5 min of rest
	RPE 3–5	RPE 3–5	RPE 3–5	RPE 3–5
	Static bike/MOTOmed	Static bike/MOTOmed	Static bike/MOTOmed	Static bike/MOTOmed

Development of exercise programme divided into strength and endurance. A,B refers to exercises specified in table. RPE, rate of perceived exertion.

**Table 2:** Participants’ baseline demographic and clinical characteristics.

Variable	n (Min–Max); Mean ± SD	Frequency (%)
Age	12 (35–66); 48.08 ± 8.55	
Years since diagnosis	12 (0.8–28); 16.65 ± 7.44	
Outbreak year	11 (0–2); 0.36 ± 0.67	
Walking time (min)	9 (0.0–120); 51.11 ± 41.06	
Sitting time (min)	9 (0–960); 466.667 ± 304.13	
**Education level**		
Primary education Secondary studies Vocational training University studies		1 (8.3%)
		1 (8.3%)
		6 (50%)
		4 (33.3%)
**Employment situation**		
Housewife Part-time employee Full-time employee Retired Permanent disability		1 (8.3%)
		1 (8.3%)
		5 (41.7%)
		3 (25%)
		2 (16.7%)
**Type of sclerosis**		
Relapsing–remitting Progressive–secondary		7 (58.3%)
		5 (41.7%)
Associated diseases		
Absence of other diseases		12 (100%)
**Outbreak intensity**		
Mild Moderate Intense No outbreaks		2 (18.2%)
		1 (9.1%)
		1 (9.1%)
		7 (63.6%)
**Fatigue medication**		
Yes No		5 (41.7%)
		7 (58.3%)
**Type of fatigue medication**		
Lioresal Lioresal + Avonex Lioresal + Rebif 44 Other medication No medication		1 (9.1%)
		1 (9.1%)
		1 (9.1%)
		2 (16.7%)
		7 (58.3%)
**Medical recommendation**		
Physical activity		6 (11.3%)
Other		1 (8.3%)
No recommendation		5 (41.7%)
**Rehabilitation**		
Yes		11 (91.7%)
No		1 (8.3%)
**Intensity rehabilitation**		
Occasional Periodic		5 (41.7%)
		7 (58.3%)
**Exercise habits**		
Occasional Regularly		2 (16.7%)
		10 (83.3%)

**Table 3:** Pre–post tDCS and exercise paired samples test: balance, functional mobility, depression, and fatigue.

	Pre-tDCS	Post-tDCS	*p*	Size Effect	Pre-Exercise	Post-Exercise	*p*	Size Effect
	Median (Range)	Median (Range)		Hedges´ g	Median (Range)	Median (Range)		Hedges´ g
**Tinetti**	12.5 (8)	14.5 (8)	0.019 *	0.632	11 (11)	14 (10)	0.004 **	0.418
**TUG**	9.46 (57.68)	7.93 (31.15)	0.012 *	0.297	10.42 (32.73)	9.02 (29.6)	0.002 **	0.262
**Beck**	15.5 (26)	11 (28)	0.013 *	0.402	15.5 (39)	9.5 (31)	0.013 *	0.540
**MFIS**	39.5 (31)	38.5 (45)	0.028 *	0.526	43 (33)	36 (52)	0.003 **	0.742

Nonparametric statistic. Wilcoxon signed-rank test. tDCS, transcranial direct current stimulation; Tinetti, Tinetti balance test; TUG, Timed Up and Go Test; Beck, Beck Depression Inventory-II; MFIS, Fatigue Modified Fatigue Impact Scale. * *p* < 0.05 and ** *p* < 0.01.

## Data Availability

The datasets used and/or analysed in the current study are available from the corresponding author on reasonable request.

## Supplementary Materials

The following supporting information can be downloaded at: https://www.mdpi.com/article/10.3390/ijerph191912747/s1, Figure S1: Changes in the presence of fatigue according to the Modified Fatigue Impact Scale (MFIS) after the application of TDCs and the exercise program.; Figure S2: Changes in the type of depression according to the Beck scale after the application of TDCs and the exercise program..

## Abbreviations

MS	Multiple sclerosis
tDCS	Transcranial direct current stimulation
NIBS	Non-invasive stimulation techniques
DLPFC	Left dorsolateral prefrontal cortex
MFIS	Modified fatigue impact scale
IPAQ-SF	International physical activity questionnaire short form
EDSS	Expanded disability status scale
BDI-II	Beck depression inventory-II
TUG	Timed Up and Go test
