# Supplementary material for: The Effect of Combining Transcranial Direct Current Stimulation Treatment and an Exercise Program on Fragility in a Population with Multiple Sclerosis: Cross-Over Design Trial
Source: Int J Environ Res Public Health. 2022 Oct 5;19(19):12747. doi: 10.3390/ijerph191912747 (PMC9566021; doi:10.3390/ijerph191912747)
Supplement: Supplementary file 1 [file ijerph-19-12747-s001.zip › ijerph-1935281-supplementary.pdf]

Figure S1. Changes in the presence of fatigue according to the Modified Fatigue Impact Scale (MFIS) after the application of TDCs and the exercise program.

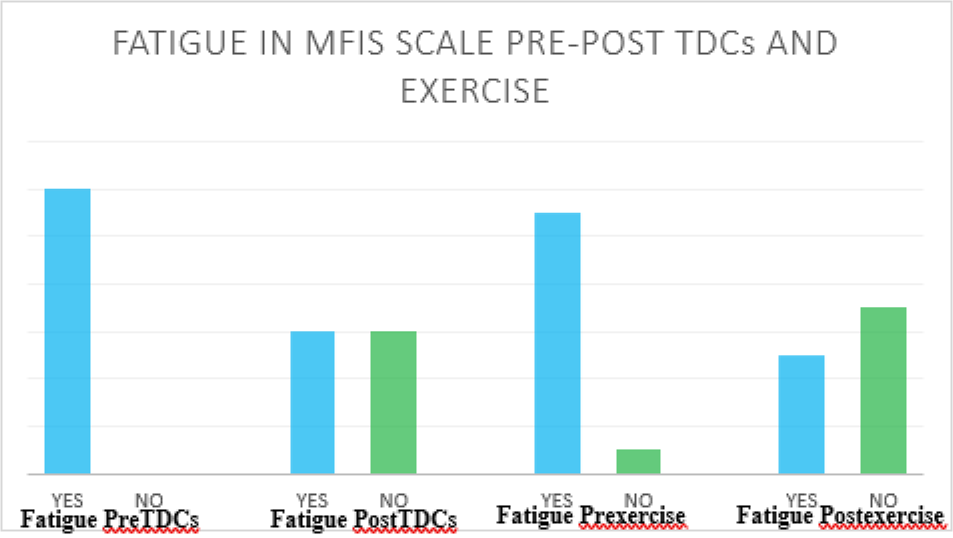

FatiguePreTDCs=Fatigue before TDCs. FatiguePostTDCs=Fatigue after TDCs. Fatigueprexercise= Fatigue before exercise. FatiguePostexercise= Fatigue after exercise.* P <0,05.

Figure S2. Changes in the type of depression according to the Beck scale after the application of TDCs and the exercise program**.**

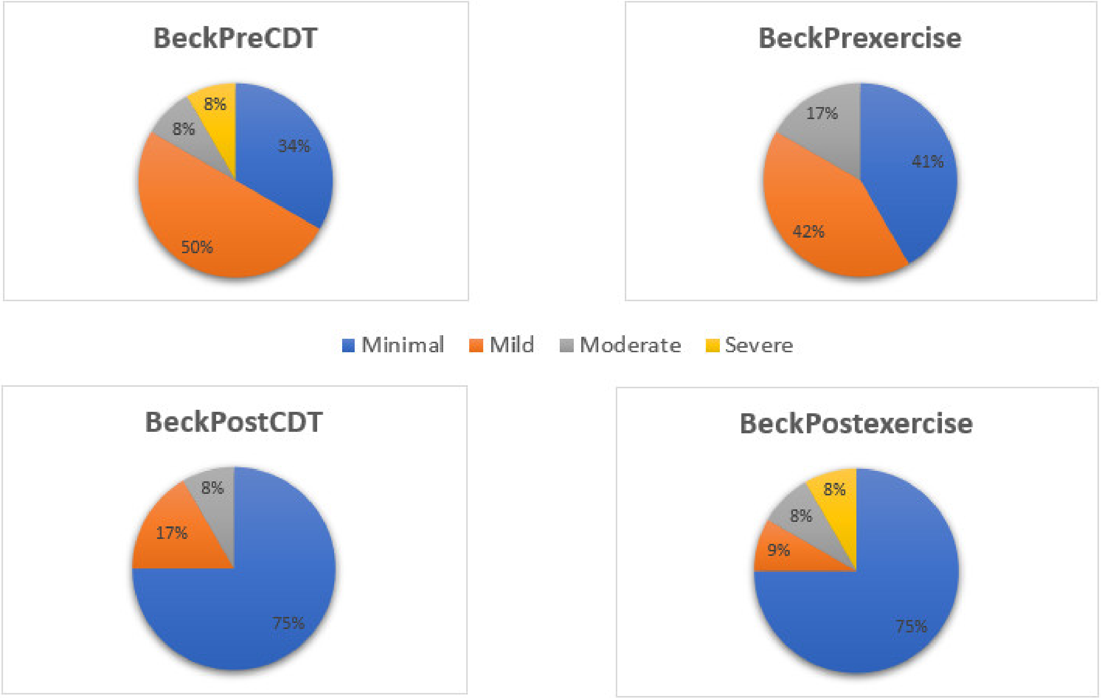

BeckPreTDCs= depression before TDCs. BeckPostTDCs=depression after TDCs. BeckPrexercise= depression before exercise; BeckPostexercise= depression after exercise
